# Supplementary material for: Metallothionein 3-Zinc Axis Suppresses Caspase-11 Inflammasome Activation and Impairs Antibacterial Immunity
Source: Front Immunol. 2021 Nov 12;12:755961. doi: 10.3389/fimmu.2021.755961 (PMC8633875; doi:10.3389/fimmu.2021.755961)
Supplement: Supplementary file 1 [file DataSheet_1.pdf]

SUPPLEMENTAL MATERIALS

TABLE S1 |

Protein-Protein Interaction Network of *Mus musculus* MT3

Sl. No	Term ID	Term Description	FDR	Protein Labels
1	GO:0060548	Negative regulation of cell death	1.12E-12	Akt1,Ppp5c,Hspb1,Sod2,Hspa8,Gsk3b,Sod1,Hsp90ab1,Hspd1,Cat,Txn1,Dnaja1,Bag1,Cdk5,Bag3,Mt3,Mt1,Ptges3,Bag5,Ar,Pgr,Hsf1,Ahr,Hspa1b
2	GO:0043067	Regulation of programmed cell death	3.01E-11	Akt1,Hspb1,Sod2,Hspa8,Gsk3b,Sod1,Hsp90ab1,Hspd1,Cat,Ppid,Dnaja1,Bag1,Vcp,Scp2,Cdk5,Bag3,Mt3,Mt1,Tspo,Bag5,Ar,Pgr,Hsf1,Nr3c1,Hdac6,Hspa1b
3	GO:0042981	Regulation of apoptotic process	1.69E-10	Akt1,Hspb1,Sod2,Hspa8,Gsk3b,Sod1,Hsp90ab1,Hspd1,Cat,Ppid,Dnaja1,Bag1,Vcp,Scp2,Cdk5,Bag3,Mt3,Mt1,Tspo,Bag5,Ar,Pgr,Hsf1,Nr3c1,Hspa1b
4	GO:0043066	Negative regulation of apoptotic process	2.34E-09	Akt1,Hspb1,Sod2,Hspa8,Gsk3b,Sod1,Hsp90ab1,Hspd1,Cat,Dnaja1,Bag1,Bag3,Mt3,Mt1,Bag5,Ar,Pgr,Hsf1,Hspa1b
5	GO:0043068	Positive regulation of programmed cell death	3.25E-08	Akt1,Sod2,Gsk3b,Sod1,Hspd1,Ppid,Dnaja1,Bag1,Vcp,Scp2,Cdk5,Tspo,Hsf1,Nr3c1,Hdac6
6	GO:2001242	Regulation of intrinsic apoptotic signaling pathway	0.0011	Hspb1,Sod2,Sod1,Dnaja1,Bag5
7	GO:0032680	Regulation of tumor necrosis factor production	0.0048	Hspb1,Hspd1,Tspo,Hsf1
8	GO:0060334	Regulation of interferon-gamma-mediated signaling pathway	0.0059	Cdc37,Hsp90ab1
9	GO:0060338	Regulation of type I interferon-mediated signaling pathway	0.0083	Cdc37,Hsp90ab1
10	GO:0032496	Response to lipopolysaccharide	0.0107	Akt1,Sod2,Hspd1,Tspo,Hsf1
11	GO:0010508	Positive regulation of autophagy	0.016	Cdc37,Gsk3b,Hdac6
12	GO:0002711	Positive regulation of T cell mediated immunity	0.0237	Hspa8,Hspd1
13	GO:0001817	Regulation of cytokine production	0.0339	Hspb1,Sod1,Hsp90ab1,Hspd1,Tspo,Hsf1
14	GO:0071222	Cellular response to lipopolysaccharide	0.0376	Akt1,Tspo,Hsf1
15	GO:0071356	Cellular response to tumor necrosis factor	0.0415	Akt1,Bag4,Cfl1

TABLE S1 | See also TABLE 1 and FILES S1 and S2 | Protein-protein interactions network of *Homo sapiens* MT3 with 50 first-shell and 50 second-shell interactors to determine functionally enriched GO BP categories using the STRING database.

FILE S1 |

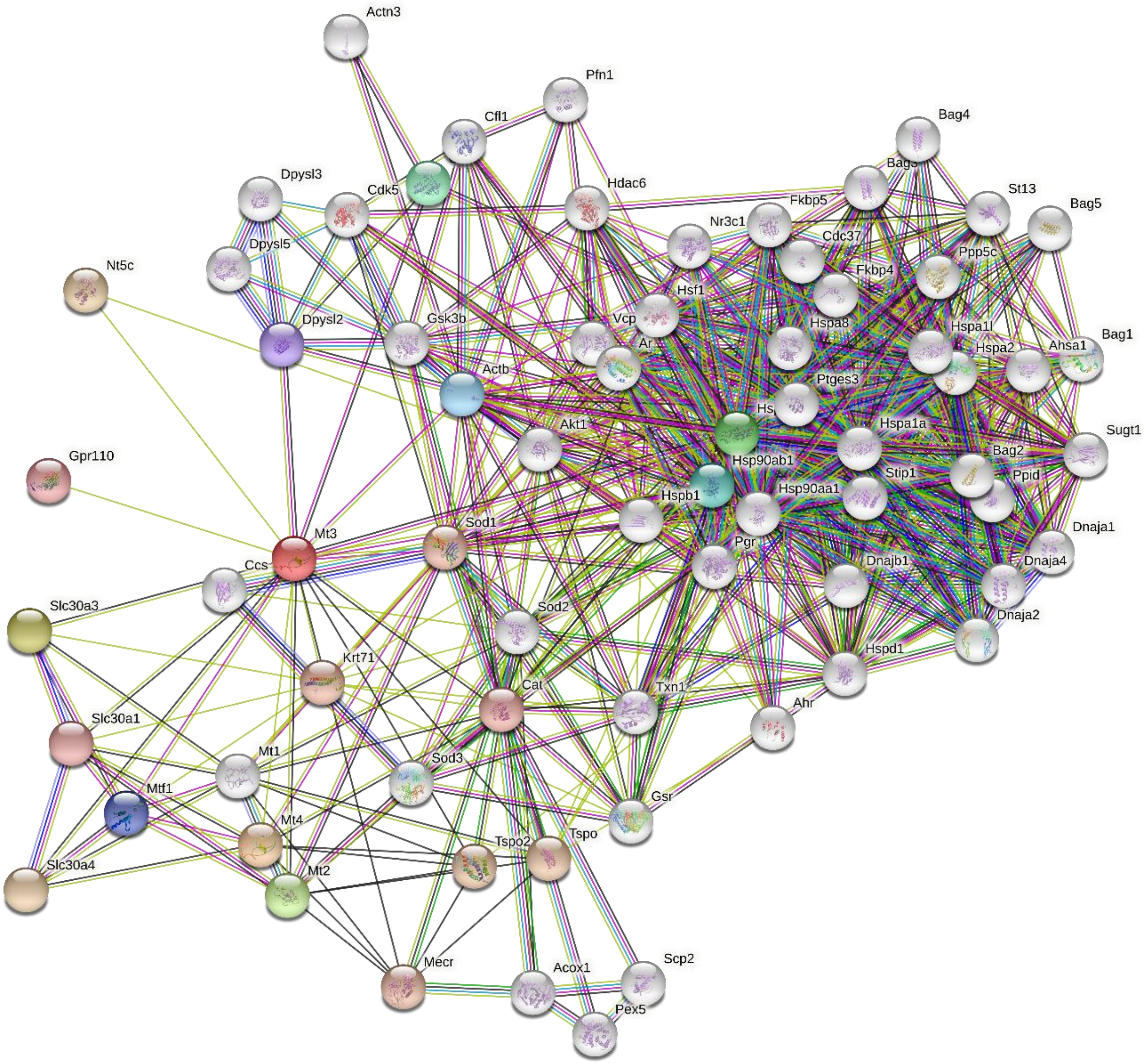
Actn3
Pfn1
Cfl1
Bag4
Bag3
Dpysl3
Cdk5
Hdac6
St13
Fkbp5
Bag5
Nr3c1
Cdc37
Dpysl5
Ppp5c
Fkbp4
Nt5c
Hsf1
Hspa8
Dpysl2
Gsk3b
Vcp
Hspa1l
Ar
Hspa2
Ahsa1
Bag1
Actb
Ptges3
Hspa1a
Akt1
Sugt1
Hsp90ab1
Bag2
Stip1
Gpr110
Ppid
Hspb1
Hsp90aa1
Sod1
Dnaja1
Mt3
Pgr
Dnajb1
Dnaja4
Ccs
Slc30a3
Sod2
Dnaja2
Krt71
Hspd1
Cat
Txn1
Ahr
Slc30a1
Mt1
Sod3
Gsr
Mtf1
Mt4
Tspo2
Tspo
Slc30a4
Mt2
Mecr
Acox1
Scp2
Pex5

FILE S2 |

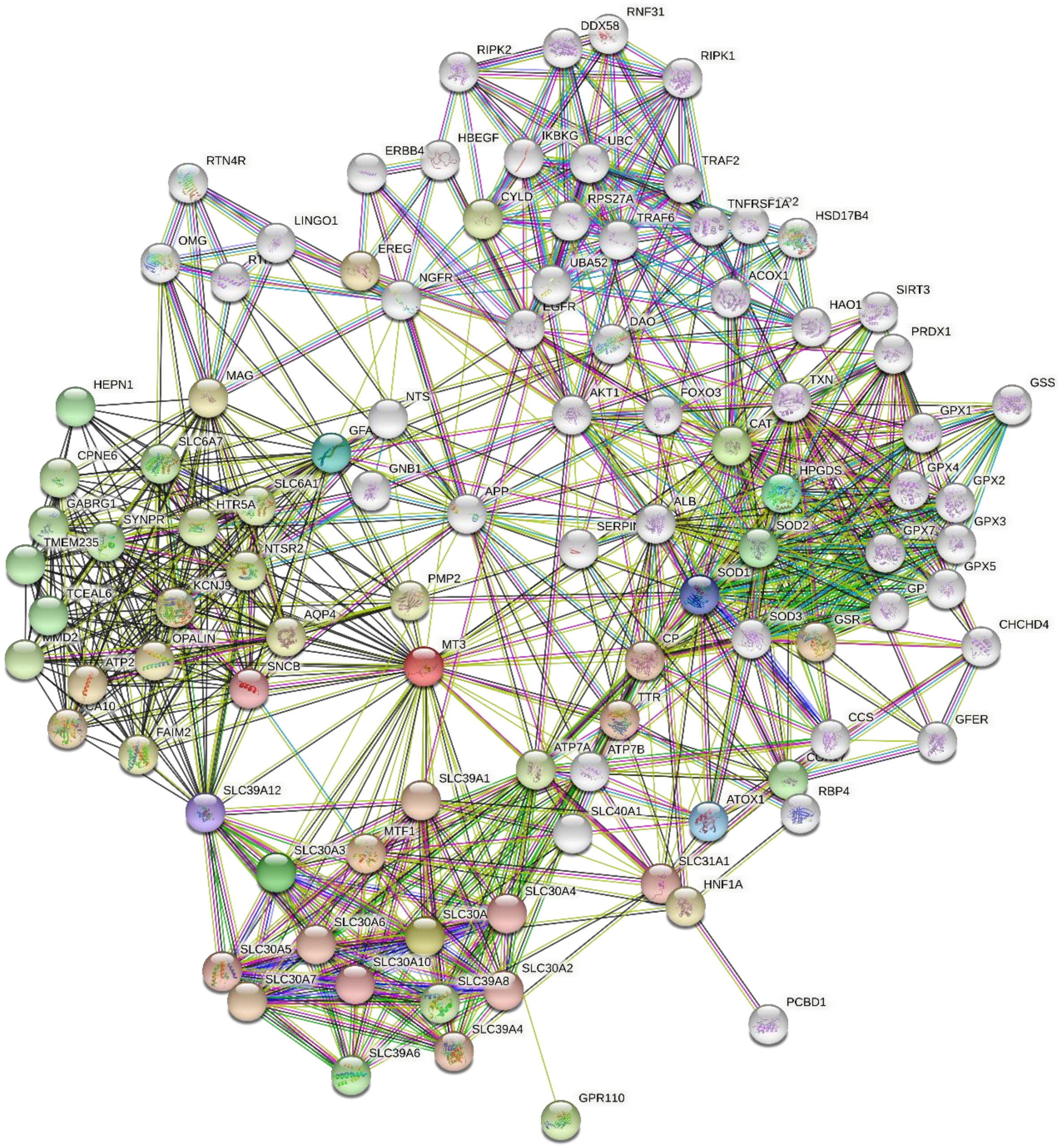

FILES S1 and S2. See also TABLE 1 and TABLE S1 | Protein-protein interaction network map of *Homo sapiens* and *Mus musculus* MT3. Protein-protein interaction

network map of MT3 in **(FILE S1)** *Homo sapiens* and **(FILE S2)** *Mus musculus* with 50 first-shell and 50 second-shell interactors showing functionally enriched gene ontology categories for biological processes (GO BP). See table 1 and table S1 for tabular representation of the GO BP categories identified using the STRING database for MT3 protein-protein network interactions in *Mus musculus* and *Homo sapiens*.

FIGURE S1

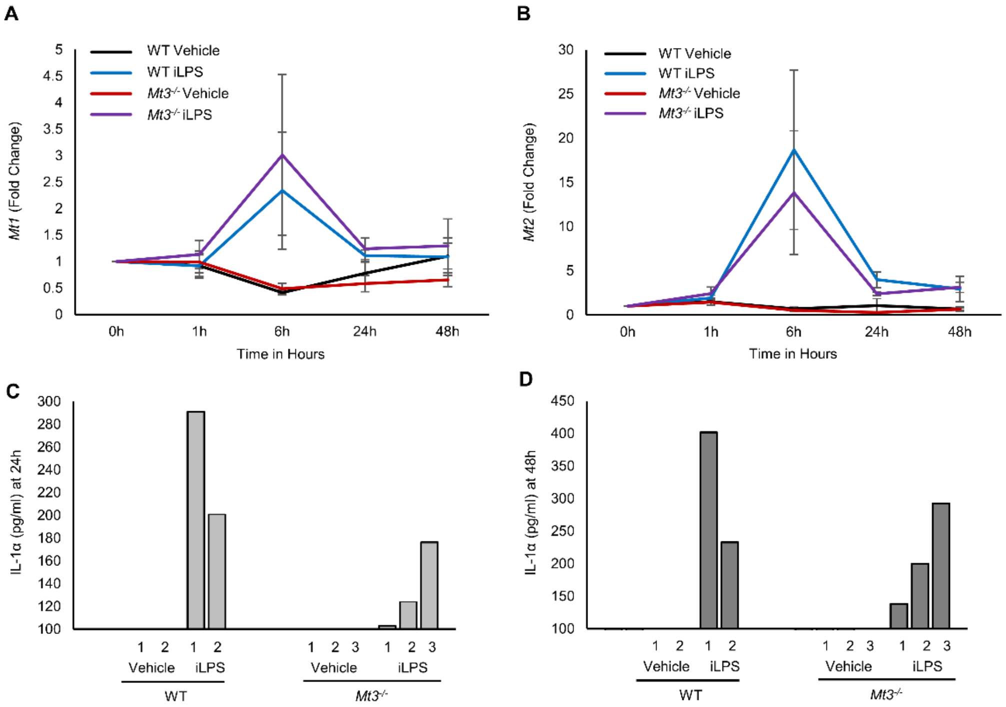

A
B
C
D
WT Vehicle
WT iLPS
Mt3-/- Vehicle
Mt3-/- iLPS
Mt1 (Fold Change)
Mt2 (Fold Change)
Time in Hours
0h
1h
6h
24h
48h
IL-1α (pg/ml) at 24h
IL-1α (pg/ml) at 48h
Vehicle
iLPS
WT
Mt3-/-

FIGURE S1 | See also FIGURE 1 | Gene expression of *Mt1* and *Mt2* and IL-1α levels in response to iLPS stimulation. qRT-PCR analysis of **(A)** *Mt1* and **(B)** *Mt2* expression over time in WT and *Mt3*$^{-/-}$ BMDMϕ stimulated with iLPS (2 μg/ml) or vehicle control. Graphs represent data from 4 independent experiments, t-test (two-tailed p-value), data are mean ± SEM. **(C, D)** IL-1α measured by ELISA in supernatants of WT and *Mt3*$^{-/-}$ BMDMϕ stimulated with 2 μg/ml iLPS or vehicle for **(C)** 24h and **(D)** 48h, 2-3 independent experiments.

FIGURE S2

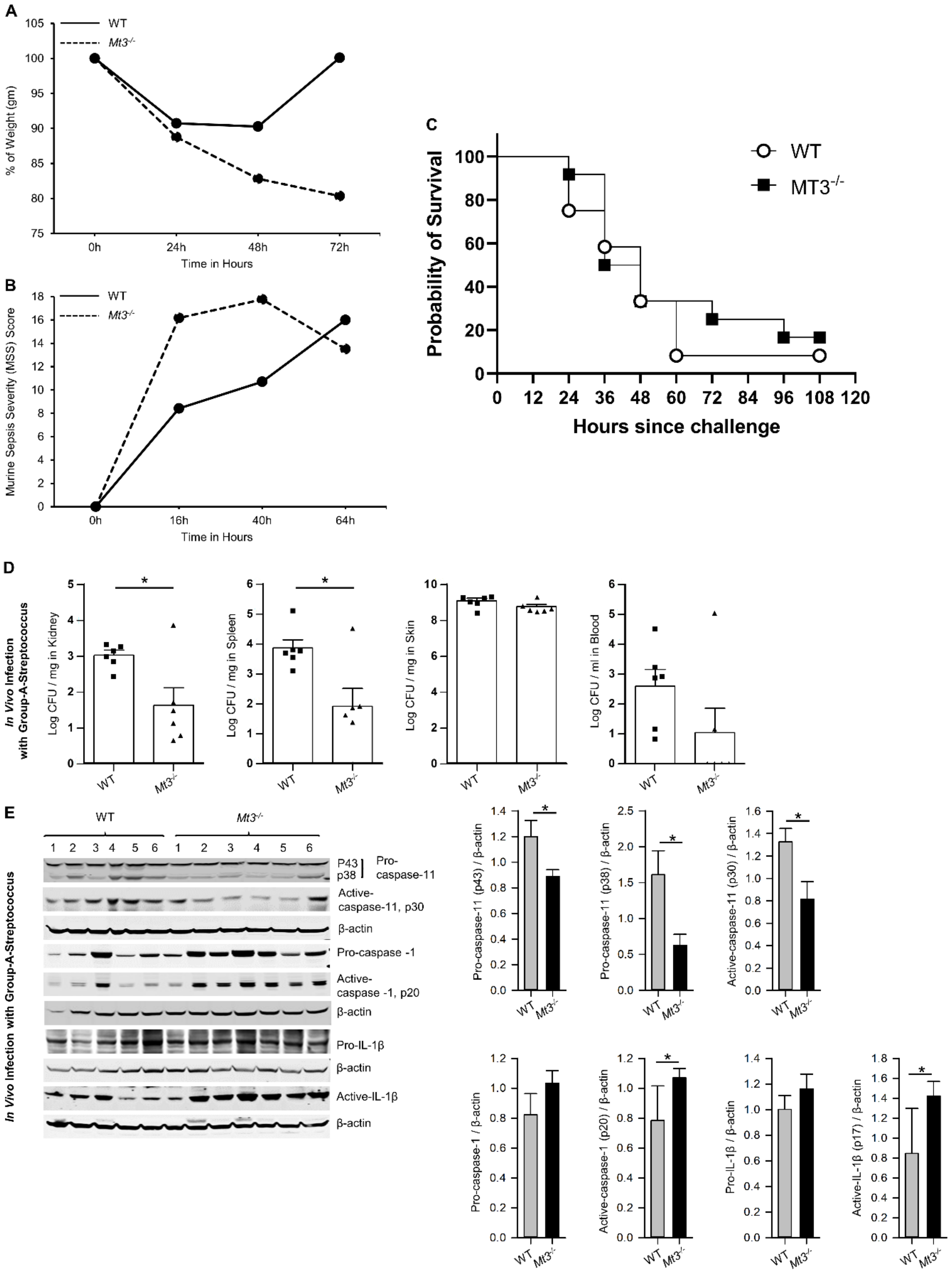
A
WT
Mt3-/-
% of Weight (gm)
Time in Hours
B
Murine Sepsis Severity (MSS) Score
C
Probability of Survival
Hours since challenge
MT3-/-
D
In Vivo Infection with Group-A-Streptococcus
Log CFU / mg in Kidney
Log CFU / mg in Spleen
Log CFU / mg in Skin
Log CFU / ml in Blood
E
P43
p38
Pro-caspase-11
Active-caspase-11, p30
β-actin
Pro-caspase -1
Active-caspase -1, p20
Pro-IL-1β
Active-IL-1β
Pro-caspase-11 (p43) / β-actin
Pro-caspase-11 (p38) / β-actin
Active-caspase-11 (p30) / β-actin
Pro-caspase-1 / β-actin
Active-caspase-1 (p20) / β-actin
Pro-IL-1β / β-actin
Active-IL-1β (p17) / β-actin

FIGURE S2 | See also FIGURE 2 | Effect of MT3 on Group A Streptococcus elimination and LPS-induced septic shock. Septic shock was induced in mice (n=12) *i.p.* with ultrapure LPS (20 mg/kg). **(A)** Weight in percentage, **(B)** MSS scoring of sepsis severity and **(C)** probability of survival was analyzed at the indicated time points. **(D)** WT and *Mt3*$^{-/-}$ mice infected *s.q.* with Group A Streptococcus (GAS5448) for 72h. Bacterial growth measured in kidney, spleen, skin and blood. CFUs were log transformed. n = 6 per group, two-tailed t-test. **(E)** Western blots of pro-caspase-11, active-caspase-11, pro-caspase-1, active-caspase-1, pro-IL1β and active-IL-1β in whole kidney homogenates. Bar graphs represent densitometric analysis of targets normalized to β-actin. n = 6 per group, two-tailed t-test, data are mean ± SEM, *p< 0.05.

FIGURE S3

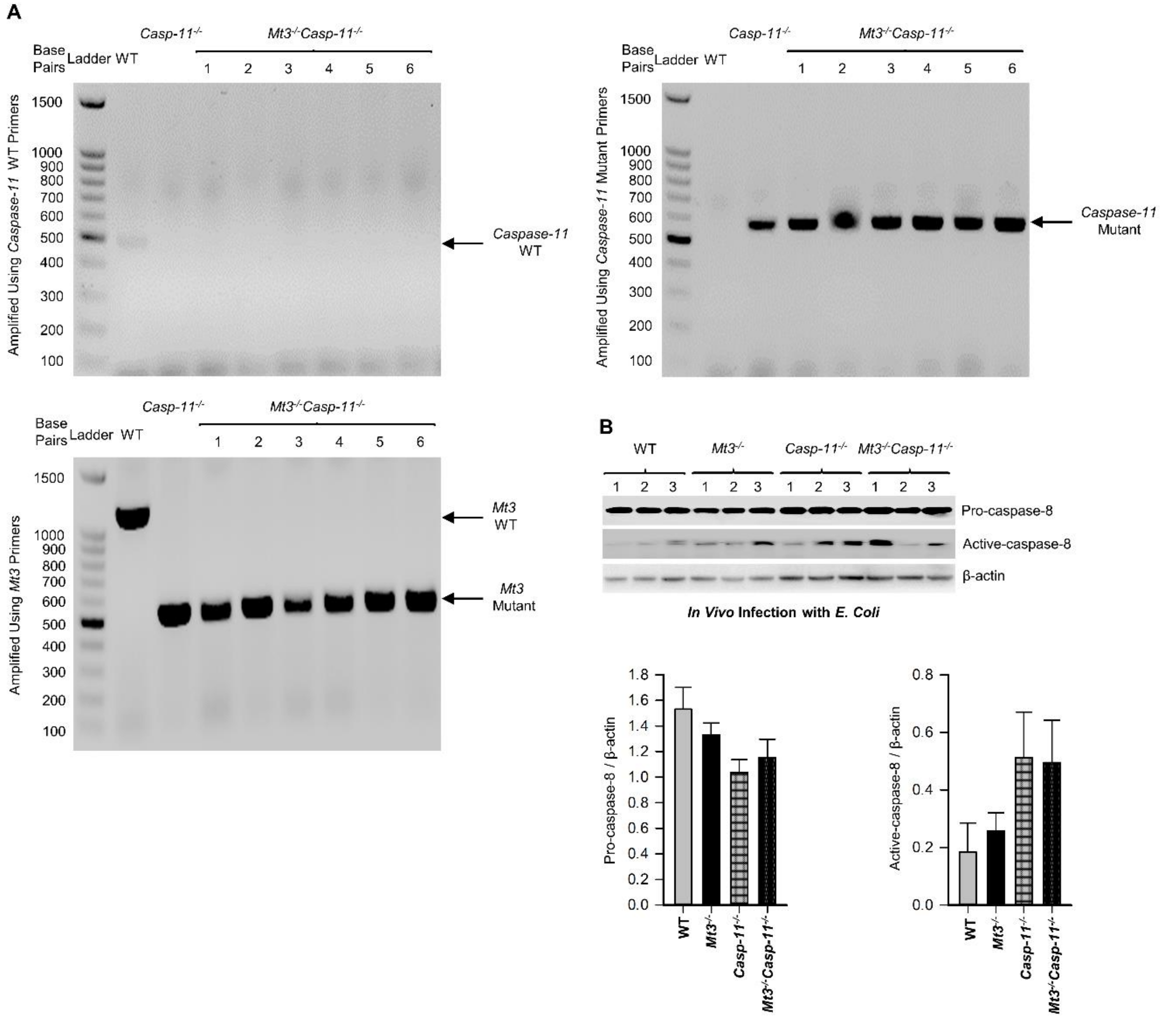

A
Casp-11-/-
Mt3-/-Casp-11-/-
Base Pairs
Ladder
WT
1
2
3
4
5
6
1500
1000
900
800
700
600
500
400
300
200
100
Amplified Using Caspase-11 WT Primers
Caspase-11 WT
Amplified Using Caspase-11 Mutant Primers
Caspase-11 Mutant
Amplified Using Mt3 Primers
Mt3 WT
Mt3 Mutant
B
WT
Mt3-/-
Casp-11-/-
Mt3-/-Casp-11-/-
Pro-caspase-8
Active-caspase-8
β-actin
In Vivo Infection with E. Coli
Pro-caspase-8 / β-actin
1.8
1.6
1.4
1.2
1.0
0.8
0.6
0.4
0.2
0.0
Active-caspase-8 / β-actin
0.8
0.6
0.4
0.2
0.0

FIGURE S3 | See also FIGURE 3 | Generation of *Casp11$^{-/-}$Mt3$^{-/-}$* mice and analysis of caspase-8 during *E. coli* infection *in vivo*. (A) *Mt3$^{-/-}$* mice crossed to *Casp4^{tm1Yuan}*/J (*Casp11$^{-/-}$*) mice to obtain mice genetically deficient in *Mt3* and *Casp-11* (*Mt3$^{-/-}$Casp11$^{-/}$*). Tail genomic DNA was amplified to confirm the deletion of *Casp-11* and *Mt3* genes by gel electrophoresis. **(B)** Western blots of pro-caspase-8 and active-caspase-8 in kidney homogenates of WT, C*asp-11$^{-/-}$*, *Mt3$^{-/-}$* and *Casp-11$^{-/-}$Mt3$^{-/-}$* mice infected *i.p.* with *E. coli* (1 X10^9 CFUs/mouse) for 6h. Bar graphs represent densitometric analysis of targets normalized to β-actin, n = 3-6 per group, one-way ANOVA, data are mean ± SEM.

FIGURE S4

A

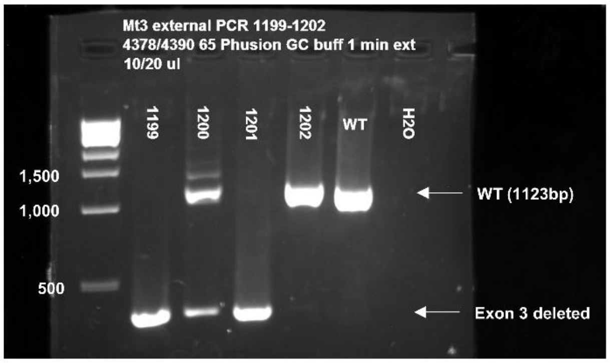

Mt3 external PCR 1199-1202
4378/4390 65 Phusion GC buff 1 min ext
10/20 ul
1199
1200
1201
1202
WT
H2O
1,500
1,000
500
WT (1123bp)
Exon 3 deleted

B

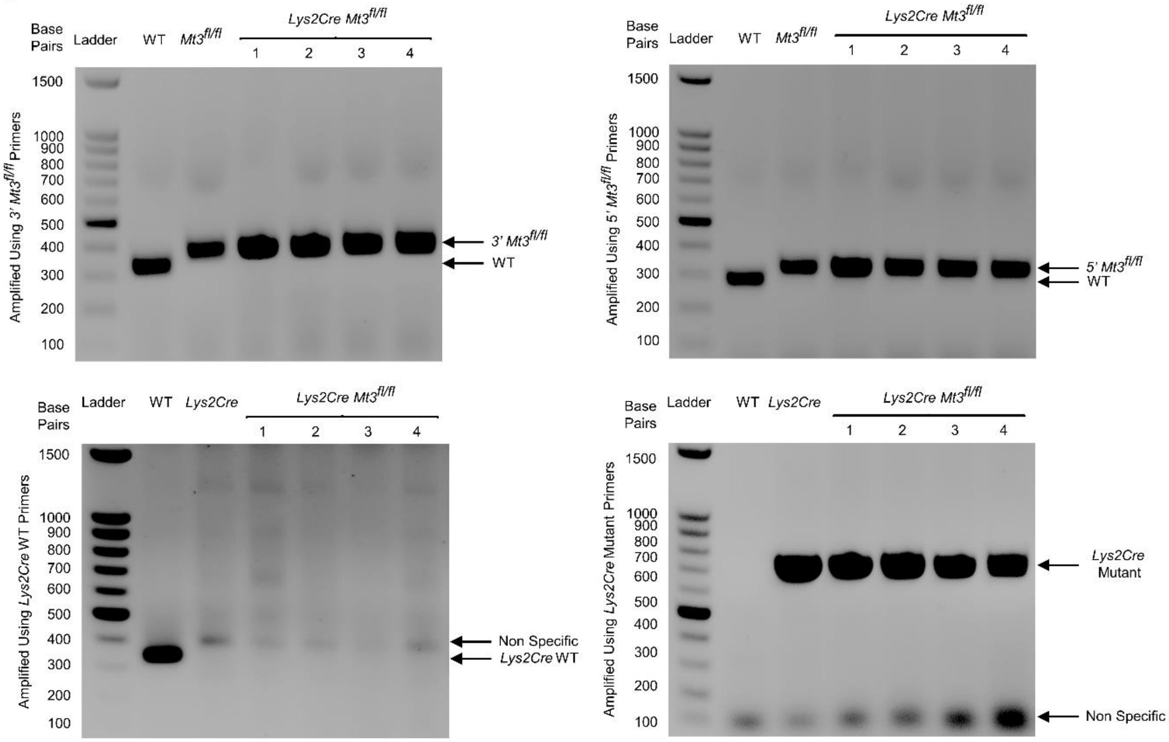

Base Pairs
Ladder
WT
Mt3fl/fl
Lys2Cre Mt3fl/fl
1
2
3
4
1500
1000
900
800
700
600
500
400
300
200
100
Amplified Using 3' Mt3fl/fl Primers
3' Mt3fl/fl
WT
Amplified Using 5' Mt3fl/fl Primers
5' Mt3fl/fl
WT
Lys2Cre
Amplified Using Lys2Cre WT Primers
Non Specific
Lys2Cre WT
Amplified Using Lys2Cre Mutant Primers
Lys2Cre Mutant
Non Specific

FIGURE S4 | See also FIGURE 4 | Generation of myeloid-MT3 deficient mice. Genotyping of WT, *Mt3*$^{-/-}$, *Lys2Cre*, *Mt3*$^{fl/fl}$ and *Lys2Cre Mt3*$^{fl/fl}$ mice. **(A)** Amplification of the *Mt3* targeted region showing homogenous *Mt3* deletion and generation of *Mt3*$^{+/-}$ heterozygous genotypes. **(B)** To confirm 5' loxp, 3' loxp and *Lys2Cre* insertion, tail genomic DNA was amplified and analyzed by gel electrophoresis using the primers detailed in the experimental procedures.

FIGURE S5

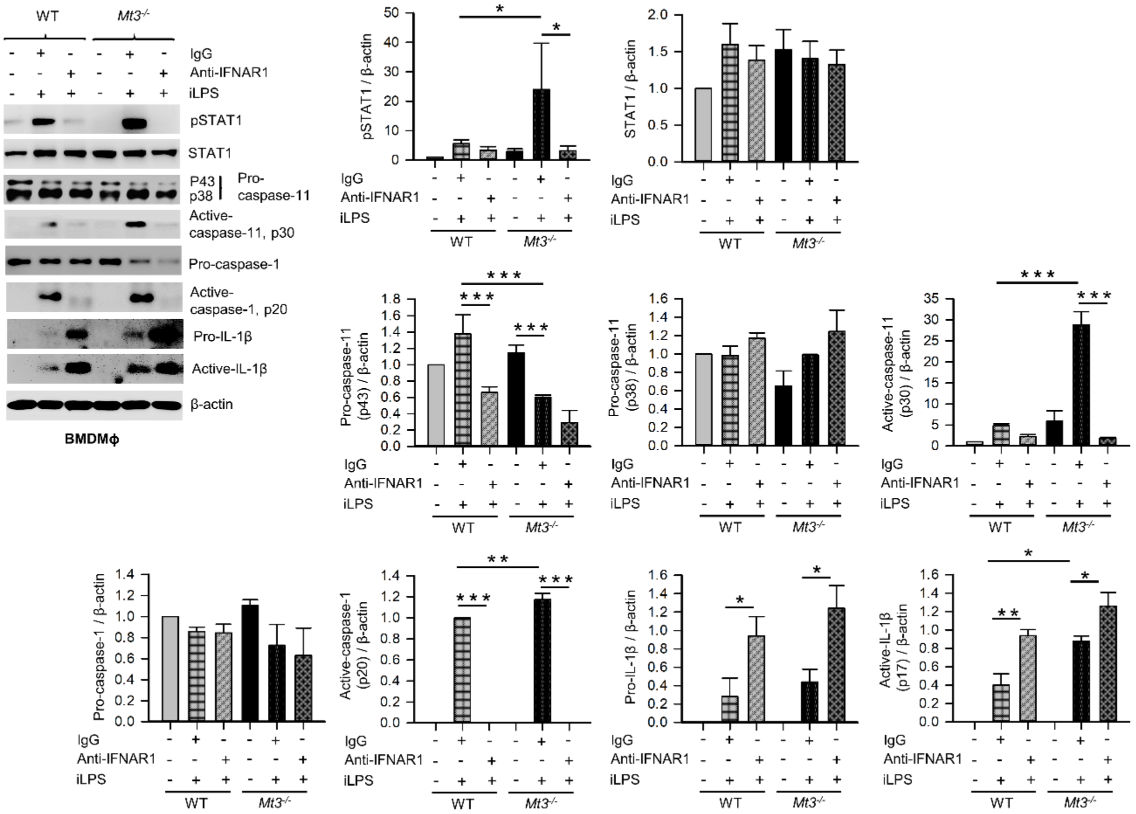

WT
Mt3-/-
IgG
Anti-IFNAR1
iLPS
pSTAT1
STAT1
P43
p38
Pro-caspase-11
Active-caspase-11, p30
Pro-caspase-1
Active-caspase-1, p20
Pro-IL-1β
Active-IL-1β
β-actin
BMDMϕ
pSTAT1 / β-actin
STAT1 / β-actin
Pro-caspase-11 (p43) / β-actin
Pro-caspase-11 (p38) / β-actin
Active-caspase-11 (p30) / β-actin
Pro-caspase-1 / β-actin
Active-caspase-1 (p20) / β-actin
Pro-IL-1β / β-actin
Active-IL-1β (p17) / β-actin

FIGURE S5 | See also FIGURE 5 | IFNAR1 blockade attenuates caspase-11 and caspase-1 activation, but augments pro- and active-IL-1β levels in BMDMϕ. WT and *Mt3*$^{-/-}$ BMDMϕ treated with monoclonal anti-IFNAR1 or IgG antibody 1h prior to and 24h after iLPS (10 μg/ml, 48h) stimulation. Western blots of pSTAT1, STAT1, pro-caspase-11, active-caspase-11, procaspase-1 and pro-IL1β in cellular extracts and active caspase-1 and active-IL-1β in the cell-free media supernatants. Bar graphs represent densitometric analysis of targets normalized to β-actin. 3 independent experiments, one-way ANOVA, data are mean ± SEM, *p < 0.05, **p < 0.01, ***p< 0.001.

FIGURE S6

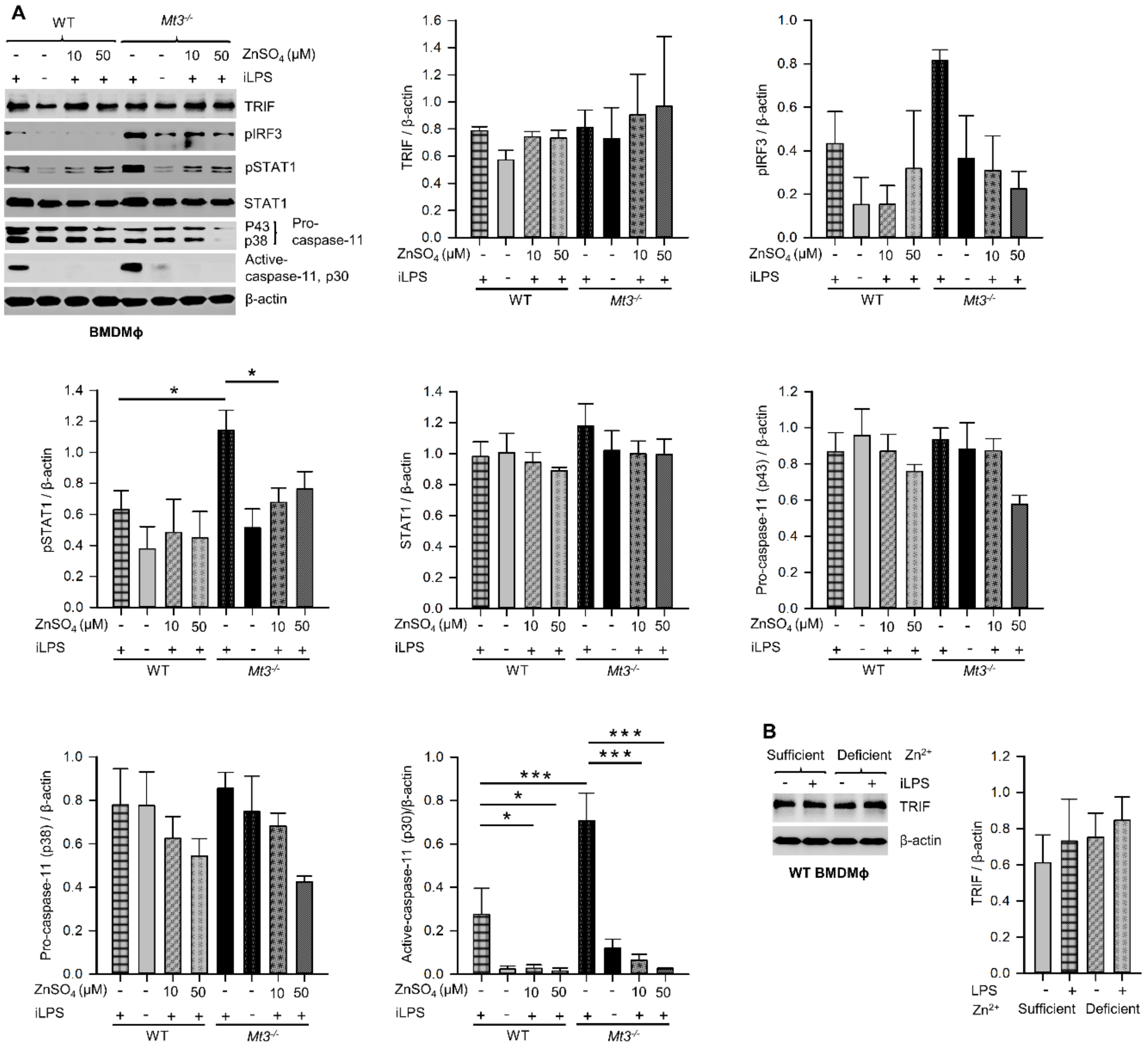

A
WT
Mt3-/-
ZnSO4 (μM)
iLPS
TRIF
pIRF3
pSTAT1
STAT1
P43
p38
Pro-caspase-11
Active-caspase-11, p30
β-actin
BMDMϕ
TRIF / β-actin
pIRF3 / β-actin
pSTAT1 / β-actin
STAT1 / β-actin
Pro-caspase-11 (p43) / β-actin
Pro-caspase-11 (p38) / β-actin
Active-caspase-11 (p30)/β-actin
B
Sufficient
Deficient
Zn2+
iLPS
TRIF
β-actin
WT BMDMϕ
TRIF / β-actin
LPS
Zn2+
Sufficient
Deficient

FIGURE S6 | See also FIGURE 6 | Zn^{2+} supplementation suppresses non-canonical inflammasome activation. (A) WT and *Mt3*$^{-/-}$ BMDMϕ exposed to the indicated doses of $ZnSO_4$ for 3h, followed by stimulation with iLPS (10 μg/ml) or vehicle for 48h. Immunoblots of TRIF, pIRF3, pSTAT1, STAT1, pro-caspase-11 and active-caspase-11 in cellular extracts. Bar graphs represent densitometric analysis of targets normalized to β-actin. 3 independent experiments, one-way ANOVA. **(B)** WT BMDMϕ stimulated with iLPS (10 μg/ml) or vehicle for 24h in Zn^{2+}-sufficient or Zn^{2+}-deficient Opti-MEM media. Immunoblot of TRIF in cellular extracts. Bar graphs represent densitometric analysis of TRIF normalized to β-actin. 3 independent experiments, one-way ANOVA, data are mean ± SEM, $^{*}p < 0.05$, $^{***}p < 0.001$.

.

FIGURE S7

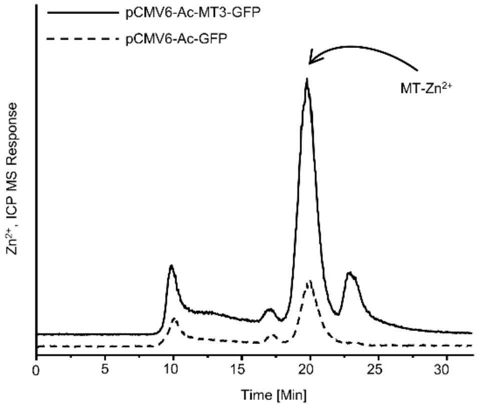

FIGURE S7 | See also FIGURE 6 | Overexpression of MT3 in *Mt1$^{-/-}$Mt2$^{-/-}$* BMDMϕ. *Mt1$^{-/-}$Mt2$^{-/-}$* BMDMϕ transfected with the *Mt3* overexpressing vector (pCMV6-Ac-MT3-GFP) or empty vector (pCMV6-Ac-GFP) control for 48 h. SEC-ICP-MS analysis of cell lysates demonstrating an increase in the MT-associated Zn^{2+} signal in cells that were transfected with the pCMV6-Ac-MT3-GFP.
